# Issues on fit-for-purpose validation of a panel of ELISAs for application as biomarkers in clinical trials of anti-Angiogenic drugs

**DOI:** 10.1038/sj.bjc.6605661

**Published:** 2010-04-20

**Authors:** K Brookes, J Cummings, A Backen, A Greystoke, T Ward, G C Jayson, C Dive

**Affiliations:** 1 Clinical and Experimental Pharmacology Group, Paterson Institute for Cancer Research, University of Manchester, Wilmslow Road, Manchester M20 4BX, UK; 2 Translational Angiogenesis Group, Paterson Institute for Cancer Research, University of Manchester, Wilmslow Road, Manchester M20 4BX, UK

**Keywords:** biomarkers, angiogenesis, ELISA, method validation, stability, platelets

## Abstract

**Background:**

Successful introduction of new anticancer agents into the clinic is often hampered by a lack of qualified biomarkers. Studies have been conducted of 17 ELISAs representing a potential panel of pharmacodynamic/predictive biomarkers for drugs targeted to tumour vasculature.

**Methods:**

The fit-for-purpose approach to method validation was used. Stability studies were performed using recombinant proteins in surrogate matrices, endogenous analytes in healthy volunteer and cancer patient plasma. The impact of platelet depletion was investigated.

**Results:**

Method validation focused on measuring precision and showed that 15 of the 17 assays were within acceptable limits. Stability at −80°C was shown for 3 months with all recombinant proteins in surrogate matrices, whereas under the same conditions instability was observed with KGF in platelet-rich and platelet-depleted plasma, and with PDGF-BB in platelet-depleted plasma from cancer patients. For measurement of extracellular circulating analytes, platelet depletion should be conducted before freezing of plasma to prevent release of PDGF-BB, FGFb and VEGF-A. A protocol was developed to remove >90% platelets from plasma requiring centrifugation at 2000 **g** for 25 min.

**Conclusions:**

These studies highlight the need for assay validation and crucial assessment of sample handling issues before commencement of biomarker analysis in clinical trials.

Angiogenesis, the formation of new blood vessels from existing vasculature, is required for tumour growth (Folkman, 1971, 1990) and is orchestrated by coordinated release of multiple signals from tumour, endothelial and stromal cells depending both on tumour type and microenvironment (Bergers and Benjamin, 2003). Following the hypothesis that inhibition of tumour angiogenesis may represent a novel approach to treat cancer (Folkman, 1971), numerous drugs targeting different facets of the angiogenic process have been developed and evaluated in clinical trials (Kerbel and Folkman, 2002). However, only a limited number are FDA-approved for treatment of cancer, including bevacizumab, a VEGF-specific blocking antibody, and the VEGF receptor tyrosine kinase inhibitors sorafenib and sunitinib (Heath and Bicknell, 2009; Jain *et al*, 2009). Many key issues remain unresolved regarding this class of drug, including the inability to stratify those patients most likely to benefit (Jain *et al*, 2009), selection of optimal dose and schedule, and how best to include vasculature-targeted agents in drug-combination regimens.

Judicious implementation of multi-modality biomarkers (tissue, imaging and blood-borne) could potentially enrich selection of likely responders and allow real-time monitoring of drug effects (Maruvada and Srivastava, 2006; Cummings *et al*, 2008). Although putative biomarkers are being increasingly applied in clinical trials of angiogenesis inhibitors, many have met with limited success (Sessa *et al*, 2008) and few, if any, qualified biomarkers exist for selecting cancer patients for antiangiogenic therapy (Sessa *et al*, 2008; Hanrahan *et al*, 2009; Jain *et al*, 2009). Imaging biomarkers have provided useful pharmacodynamic information (O’Connor *et al*, 2009), but these are expensive, restricted to clinical trial sites with specialised expertise and less amenable to comprehensive serial sampling than blood-based analyses.

Studies using circulating biomarkers of angiogenesis have produced conflicting and often confusing results and this may reflect complex biology, differences in antibody versus small-molecule drugs and/or lack of assay validation (Twardowski *et al*, 2008; Backen *et al*, 2009; Treiber *et al*, 2009). Certainly, assay validation data in the public domain are scarce. Few studies have been reported where comprehensive, qualified panels of circulating factors associated with angiogenesis have been implemented and consequently any additional power of a large biomarker panel to predict or report drug effect is unclear. Such studies of biomarker candidates measured with validated assays are now warranted to discover and qualify optimised panel(s) of biomarkers and thus improve the use of antiangiogenic drugs for cancer treatment.

Method validation constitutes a crucial component in biomarker research, and it is often the case that a biomarker can fail in the clinic not because of the underlying scientific rationale but rather from poor assay choice and lack of robust validation (Pepe *et al*, 2001; Bast *et al*, 2005; Wagner *et al*, 2007). In this study, validation studies were conducted on 17 ELISAs representing a panel of potential pharmacodynamic and/or predicative biomarkers pertaining to tumour vasculature-targeted drugs. The ‘fit-for-purpose’ approach to biomarker method validation was adopted, including a faster track element used to explore assay capabilities, with consideration to issues of stability, impact of platelets, sample handling and method change (Lee *et al*, 2005, 2006).

## Materials and methods

### Single-plex sandwich ELISAs

Seventeen ELISA kits (Quantikine Human - Sandwich ELISA – Immunoassay; R&D Systems Europe Ltd, Abingdon, UK) representing a comprehensive panel of putative biomarkers of angiogenesis were evaluated (see Table 1 for abbreviations). Kit expiry dates were logged and kits were stored at 2–8°C before use. Once opened, the kit contents were stored at 2–8°C for up to 1 month. The ELISAs were run essentially according to the manufacturer's instructions, with the exception that plates were all washed using an automated plate washer (Columbus, Tecan Trading AG, Switzerland) and a standard ELISA wash buffer was obtained from PEVIVA (Bromma, Sweden). ELISA plates were analysed using a Dynex MRXII reader using the Revelation software (version 4.03). Performance of the plate reader was qualified each month using a Dynex calibration plate certified by the UK National Physics Laboratory (NPL, Rugby, England). Lyophilised Quantikine ELISA Kit Controls (QCs; R&D systems) were stored at 2–8°C before reconstitution at the recommended concentration using the appropriate Calibrator Diluent pertaining to each analyte. All QCs were discarded immediately after use.

### Multiplex ELISA assays

Mindful of minimising patient sample volume, the Searchlight Multiplex ELISA system was also included (Aushon BioSystems, Boston, MA, USA) allowing further validation and a more comprehensive evaluation of sample stability. Two multiplex ELISAs were used: a 5-plex comprising VEGFR1, VEGFR2, IL8, keratinocyte growth factor (KGF) and PIGF, and a 4-plex comprising platelet-derived growth factor (PDGF-BB), HGF, FGFb and VEGF-A. Plex expiry dates were logged and plexes stored at 2–8°C before use. Both assays were used according to the manufacturer's instructions and had been subjected to extensive method validation by our laboratory before this study (Backen *et al*, 2009). The plexes were imaged using a SearchLight Plus CCD (charge-couple device) Imaging System and images were analysed using the SearchLight Array Analyst software (version 2.2). The software and camera were subjected to installation, operational and performance qualification (IQ, OQ and PQ) for use in clinical trials (Backen *et al*, 2009). PQ was performed monthly using a Glowell low-light imaging standard (UVP, Cambridge, UK; catalogue number GLO-014) and calibrated annually by SP Technical Research Institute of Sweden. The standards for each of the nine multiplexed angiogenesis biomarkers (supplied with the kits) were stored, diluted and handled as recommended in the manufacturer's instructions.

### Fit-for-purpose validation of single-plex ELISA assays

**Figure 1:**
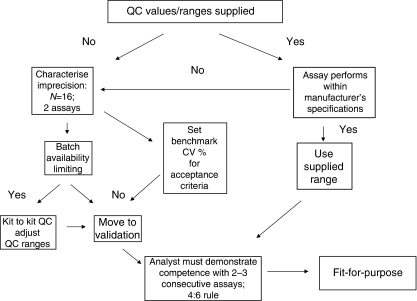
Fit-for-purpose biomarker ELISA validation for use in clinical trials. Fit-for-purpose biomarker method validation was essentially a demonstration that a commercially available assay consistently performs within specification (either manufacturers or set in-house) using QCs before patient sample analysis and consisted of three stages. In the first stage the precision (as % CV) in the QCs was determined experimentally. In stage-2, a target CV acceptance limit was set against which the performance of future assays was evaluated. Stage-3 required that 2–3 additional assays fell within these target CVs for the QCs, to consider the assay valid for analysis of clinical trials samples. In the light of present data enhancement to this scheme is now recommended (see Discussion).

The performance of the 17 different single-plex ELISA assays in terms of precision was determined using quality-control (QC) samples at three different concentrations corresponding to the low end, middle and top end of the calibration curve for each analyte as detailed in Figure 1.

### QC and batch-to-batch variation

Throughout the duration of the studies, validation experiments were performed upon introduction of either a new batch of kit(s) or a new batch of QCs (Figure 1). In either case eight replicates of high, medium and low QCs were run; the mean value, standard deviation (s.d.) and coefficient of variation (CV) were determined and compared with values obtained with the previous batches of ELISA kits or QCs. A difference of <25% from the mean value was required to accept the new batch.

### Stability of recombinant protein standards in surrogate matrices

Lyophilised carrier-free recombinant (r) protein standards were stored at −20°C before reconstitution in a surrogate matrix for the stability studies. The reconstituted r-proteins were spiked at a mid-range concentration into porcine/bovine plasma and serum stocks (Scipac, Sittingbourne, UK) and 300-*μ*l aliquots were stored at room temperature (RT), 4°C, −20°C or −80°C for up to 12 months. At defined intervals samples were retrieved for analysis in duplicate by single-plex ELISA, with instability being defined as a significant reduction in concentration (⩾25%) occurring between two time points. Freeze–thaw samples were analysed in triplicate before freezing to −80°C and after defrosting at RT for up to three cycles. Freeze/thaw intolerance was defined as a significant reduction in concentration of ⩾25%.

### Stability of endogenous angiogenesis analytes in human plasma collected from healthy volunteers

Normal human plasma (prepared in EDTA) from two healthy volunteers was obtained from Scipac Ltd. Upon receipt the plasma samples were analysed by multiplex ELISA using eight replicates; they were then stored in aliquots at −80 °C for 8 months before re-analysis by multiplex ELISA (*n*=8). Instability was defined as a significant reduction in concentration (⩾25%) between the two time points.

### Stability of endogenous angiogenesis analytes in plasma collected from colorectal cancer patients

All studies using patient samples were performed under ethical committee approval and all patients provided informed consent (REC Ref: 06/Q1406/117). Plasma samples for stability studies were obtained from five patients with colorectal cancer. Four aliquots were prepared from each patient sample by different centrifugation protocols, including platelet-depleted plasma (see below); they were analysed in triplicate upon receipt by multiplex ELISA and stored at −80°C for 3 months before re-analysis by multiplex ELISA. Instability was defined as a significant change in concentration (⩾25%) between the two time points.

### Effect of platelet inclusion/exclusion and freeze thaw on angiogenesis analyte concentration determined in plasma collected from colorectal cancer patients

To evaluate the impact of platelets on the measurement of the panel of angiogenesis-related analytes by ELISA, blood was collected from 20 patients with metastatic colorectal cancer who were receiving conventional chemotherapy at The Christie NHS Foundation Trust (Manchester, UK). A 20-ml volume of venous blood was withdrawn from each subject and transferred to an EDTA vacutainer and labelled as the whole-blood (WB) specimen. Aliquots of the WB specimen were retained for platelet count by the Haematology Department of The Christie. The WB specimen was centrifuged at 2000 **g** for 10 min at RT to separate the plasma (A). Aliquots of sample-A were retained for platelet counts or stored at −80°C for both single- and multiplex ELISA analysis. The remainder of sample-A was divided into three aliquots, each to be centrifuged further at RT by one of three procedures: 2000 **g** for 15 min (A+B), 2000 **g** for 20 min (A+C) or 10 000 **g** for 10 min (A+D). Aliquots of each were retained for platelet count or stored at −80°C for both single- and multiplex ELISA analysis. A final confirmation study was performed in the same way using blood from three healthy volunteers comparing WB and sample-A (2000 **g** for 10 min) using a single centrifugation of WB at 2000 **g** for 25 min (E). The effect that freezing plasma sample-A to −80^o^C before re-centrifugation (at 2000 **g** for 15 min) had on angiogenesis analyte concentrations was also investigated by multiplex ELISA.

## Results

### Fit-for-purpose validation of single-plex ELISA assays

The process developed to validate the panel of 17 ELISAs using QCs is described schematically in Figure 1. The first stage was to characterise assay performance (mean value±precision) by analysis of 16 replicates of each of three different QCs representing high-, mid- and low-range concentrations on the calibration curve. To complete this stage at least two separate assays were performed. The second stage was to set ‘benchmark’ acceptance limits against which the performance of subsequent assays was evaluated. Changes in batches of ELISA kits or QCs required batch-to-batch analysis (*n*=8 replicates) and often resulted in adaptation of acceptance limits. Finally, in stage three an analyst was required to show in 2–3 consecutive assays that all three QCs continued to fall within their acceptance limits, using a 4 : 6 rule.

Results on the above validation process for the 17 ELISAs are reported in Table 1. Precision was always less than 20% and in most cases less than 15% for each QC (Table 1); thus the ‘benchmark’ acceptance limit of 20% was set around the mean experimental value. In the subsequent 2–3 assays only 15 of the 17 assays showed consistency. VEGF-C and KGF failed validation at this stage and were not taken forward for further validation experiments, including analysis of cancer patients’ samples.

### Stability studies of angiogenesis analytes

Stability studies were conducted in three stages: first by adding a known concentration of a r-protein to a surrogate matrix; second by measuring endogenous angiogenesis analytes in plasma collected from healthy volunteers and third by re-analysis of plasma samples taken from cancer patients. The stability of r-proteins spiked into plasma and serum, and stored at RT, 4°C, −20°C and −80°C, is reported in Table 2. With the exception of PlGF stored at 4°C, there was no difference in stability profiles between serum and plasma. All analytes were unstable at RT and 4°C, with PlGF being particularly unstable at RT. The data suggest that storage of samples at 4°C for longer than 7 days is not recommended in general, and specifically, that PDGF-BB, PlGF and VEGF-A should be kept at 4°C for no longer than 24 h (Table 1). With the exception of SDF-1*α* at −80°C and PlGF at −20°C, all other analytes investigated (nine in total; Table 1) were stable for at least 3 months at both temperatures and for three freeze–thaw cycles (data not shown).

**Figure 2:**
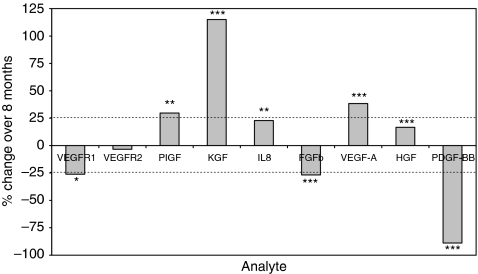
Stability of endogenous angiogenesis analytes in healthy-volunteer plasma. Plasma from healthy volunteers was analysed by multiplex ELISA (*n*=8 replicates per time point) before and after storage at −80°C for 8 months. Instability was defined as a significant change in concentration ⩾25% between the two time points (Student's *t*-test: ^*^P<0.05; ^**^P<0.01 and ^***^P<0.001). The greatest degree of instability was shown with PDGF-BB (89% decrease) and KGF (115% increase). Smaller (>25% but <38%) but significant changes also occurred with VEGFR1, FGFb, PlGF and VEGF-A.

Endogenous analytes were measured in pooled healthy volunteer plasma (*n*=8 replicates), before storage at −80°C and after 8 months. Statistical differences between these two time points were determined by Student's *t*-test. However, an acceptance limit for significance was imposed at a 25% increase or decrease in concentration to take account of assay variability (see Figure 2). Under these criteria, small (>25% but <38%) but significant changes occurred with VEGFR1, FGFb, PlGF and VEGF-A (Figure 2). The greatest degree of instability was recorded with KGF (115% increase) and PDGF-BB (−89% decrease).

**Figure 3:**
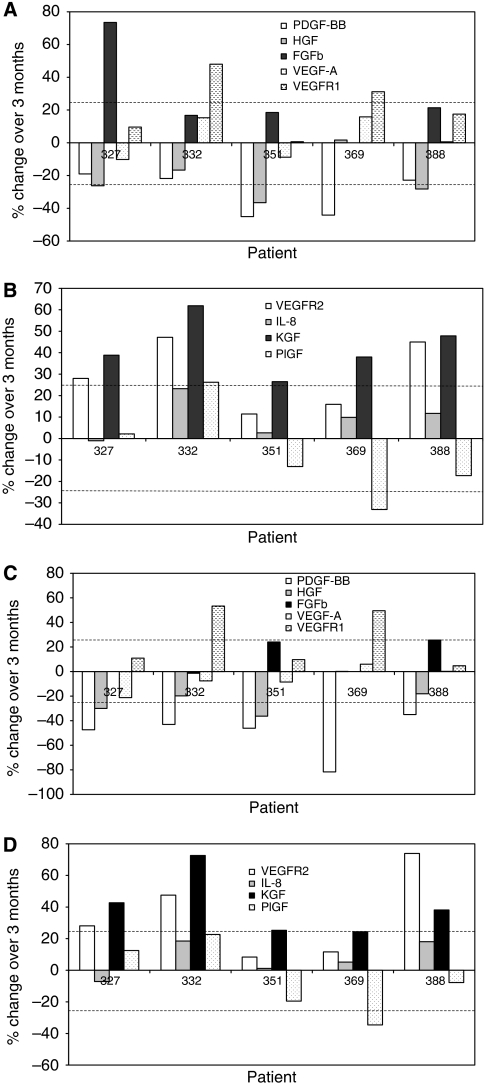
Stability of endogenous angiogenesis analytes in both platelet-rich and platelet-depleted plasma in cancer patients. Plasma from five different colorectal cancer patients was analysed by multiplex ELISA before and after storage at −80°C for 3 months. The platelet-rich and platelet-depleted samples were produced as described in Figure 4. Instability was defined as a significant change in concentration ⩾25% between the two time points (Wilcoxon signed-rank test). The Wilcoxon signed-rank test assessed consistent trends within the group of five patients. Only in the case of KGF was consistent instability (manifest as an increase in concentration) observed in both platelet-rich (*P*<0.0001) and platelet-depleted (*P*=0.002) plasma, whereas consistent reduction in PDGF-BB concentrations was recorded in platelet-depleted plasma (*P*=0.006). Sporadic changes or ⩾25% were measured in individual patients with a number of other analytes such as a 74% increase in FGFb in platelet-rich plasma in patient-327 and a 74% increase in VEGFR2 in platelet-depleted plasma in patient-388. For PDGF-BB, HGF, FGFb, VEGF-A and VEGFR1 see panel **A** for platelet-rich plasma and panel **C** for platelet-depleted plasma, and for VEGFR2, IL8, KGF and PlGF see panel **B** for platelet-rich plasma and panel **D** for platelet-depleted plasma.

To evaluate stability in cancer patients’ plasma, angiogenesis analytes were measured in four replicate plasma samples collected from five different patients: a study design that allowed between-patient comparison to be made in platelet-rich (Figure 3A and B) and platelet-depleted plasma (Figure 3C and D). Stability was assessed at −80°C by analysing the samples immediately after storage and 3 months later. Instability is defined as above for healthy plasma except that statistical significance was evaluated by the Wilcoxon signed-rank test.

After 3 months, a significant change (increase) in concentration was detected consistently in all five patients with only one analyte in both platelet-rich (KGF, *P*<0.0001; Figure 3B, 26–62%) and platelet-depleted plasma (KGF, *P*=0.002; Figure 3D, 24–73%). However, a consistent reduction in PDGF-BB concentrations was also recorded in platelet-depleted plasma (*P*=0.006; Figure 3C, 35–82%). With all the other seven analytes studied, more sporadic changes were evident both in platelet-rich and platelet-depleted plasma. Nonetheless, these data show that unexpected fluctuations in the concentrations of angiogenesis analytes can occur in individual patients: for example a 74% increase was seen in FGFb in patient-327 (Figure 3A) and a 74% increase was observed in VEGFR2 in patient-388 (Figure 3D), even after 3 months of storage at −80°C. Importantly, these changes would not have been predicted from the stability studies using r-proteins in surrogate matrices. These data indicate that r-protein and surrogate matrices are not sufficiently predictive of the clinical situation.

### Effect of platelet inclusion or removal, and freeze–thaw, on angiogenesis analyte concentrations measured in cancer patients’ plasma

**Figure 4:**
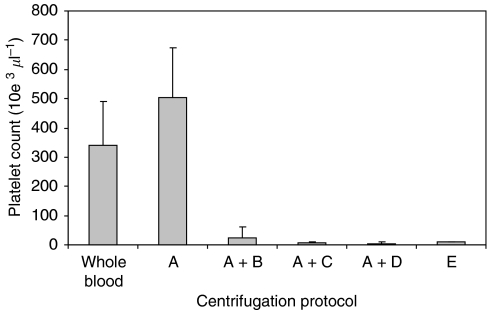
Platelet count in plasma prepared using differing centrifugal techniques. Platelet counts were initially measured in WB collected from 20 colorectal cancer patients and in plasma (A) produced after centrifugation at 2000 g for 10 min. Plasma (A) was then subjected to re-centrifugation to produce three further samples: 2000 g for 15 min (A+B), 2000 g for 20 min (A+C) or 10 000 g for 20 min (A+D). The platelet count after spinning using method A+B was significantly (*P*<0.05, ANOVA) different from spin A. There was no significant difference between spin A+B, A+C and A+D. Blood subjected to a single centrifugation step at 2000 g for 25 min (E) yielded a platelet count significantly (*P*<0.05) different from A but not A+B/C/D, indicating a one-step method to effectively deplete platelets in a clinical setting.

Cancer patients’ plasma was centrifugated in stages to determine the minimum duration and optimal speed to remove platelets effectively from plasma. The protocols adopted in this study yielded four different plasma samples: each was subjected to a standard procedure, but three samples received an additional spin of either increasing duration or centrifugal force (see Methods and Figure 4). The standard procedure adopted to separate plasma, 2000 **g** for 10 min at RT, did not significantly deplete platelets as compared with that in WB. However, further centrifugation at 2000 **g** for 15 min at RT effectively removed 93% of platelets (*P*<0.05: ANOVA, corrected using Bonferroni Multiple Comparison Test) (Figure 4). A spin at 2000 **g** for 20 min increased this value to 99%, although the difference between the two procedures was not significant (ANOVA). Likewise, high-speed centrifugation at 10 000 **g** for 10 min offered no further advantage (ANOVA) (Figure 4). A subsequent study confirmed that centrifugation of WB at 2000 **g** for 25 min (E; *P*<0.05: ANOVA, corrected using Bonferroni Multiple Comparison Test) was an equally effective protocol for platelet removal, without recourse to high-speed centrifugation equipment or a two-step preparation method.

**Figure 5:**
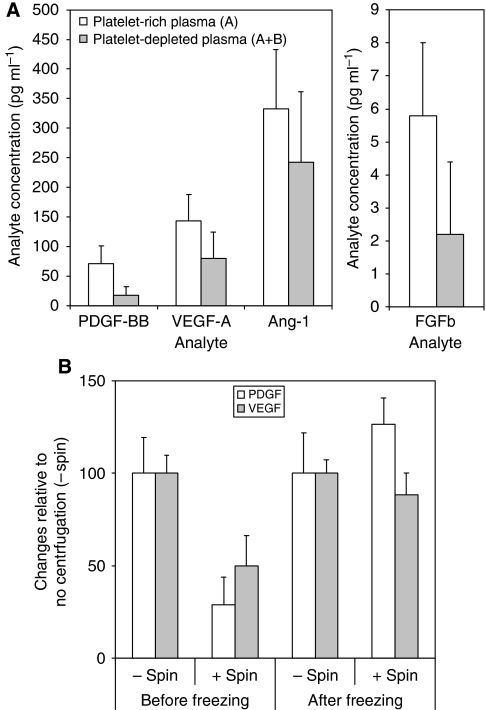
Effect of platelet inclusion or removal and freeze–thaw on the concentration of angiogenesis analyte measured in cancer patients’ plasma. (**A**) Angiogenesis analyte concentrations in five different colorectal cancer patients were initially measured in plasma prepared by centrifugation at 2000 g for 10 min (A) and then re-measured after re-centrifuged at 2000 g for 15 min (A+B) to deplete platelets by >90%. Platelet depletion resulted in a significant (*P*<0.05, ANOVA) decrease in concentration in the case of PDGF-BB, FGFb and VEGF-A but not with Ang-1 (and SDF-1*α*, not shown), although the latter two analytes are thought to be sensitive to platelets. (**B**) Although both PDGF-BB and VEGF-A levels were significantly reduced by re-centrifugation of plasma resulting in platelet depletion this was only the case before freezing the plasma. However, if the re-centrifugation step was performed after freezing the plasma no significant reduction of PDGF-BB and VEGF-A occurred.

Of the analytes investigated, removal of platelets reduced significantly the plasma concentrations of PDGF-BB (mean reduction in five different patients of 77%), FGFb (63%) and VEGF-A (43%) (Figure 5A; *P*<0.05: ANOVA, corrected using Bonferroni Multiple Comparison Test). Although there was a trend towards reduction in Ang-1 concentrations upon removal of platelets (Figure 5A), this did not reach statistical significance. SDF-1*α* concentration in plasma has also been reported to be affected by the presence of platelets, but in this study removal appeared to have little effect (data not shown). Linear regression analysis showed a strong correlation between platelet numbers and plasma concentrations of PDGF-BB (*P*=0.0002), FGFb (*P*=0.0001) and VEGF-A (*P*=0.042).

The data show that removal of platelets reduced the plasma concentration of certain angiogenesis-associated factors, if the platelets were removed before freezing plasma samples. Once plasma samples were frozen and platelets presumably ruptured, then centrifugation was without effect on angiogenesis analyte concentrations (see Figure 5B). The results of these studies provide data to guide decisions concerning platelet removal protocols and highlight the importance of documenting the presence or absence of platelets to optimise data analysis.

## Discussion

With the eventual objective of qualifying biomarkers to facilitate the clinical development of drugs targeted to the tumour vasculature, a panel of ELISAs for circulating angiogenesis associated factors was validated (see Table 1) (Jain *et al*, 2009). Specifically, a ‘fit-for-purpose’ method validation was undertaken with the aim of identifying and minimising variability associated with the sample analysis cycle, often the cause of biomarker failure in the clinic (Pepe *et al*, 2001; Bast *et al*, 2005; Wagner *et al*, 2007). In the UK and Europe method validation is a requirement of the Clinical Trials Regulations (Cummings *et al*, 2008). Thus, in this study our focus was to develop a strategy to validate a large panel of ligand binding (sandwich ELISA) assays (LBAs) (Shah, 2007) and to characterise sample handling issues associated with analysis of circulating soluble angiogenesis regulators such as stability and influence of platelets (Nayeri *et al*, 2002; Findlay, 2009; Mahler *et al*, 2009).

The strategy adopted (Figure 1) used QCs to monitor performance (Lee *et al*, 2005, 2006). Preliminary studies were conducted to characterise the error associated with each assay to set realistic acceptance criteria against which to judge the performance of subsequent assays, rather than imposing rigid guidelines in advance such as in bioanalytical method validation (Shah *et al*, 1991, 2000). In this context, ‘fit-for-purpose’ for use in clinical trials was defined essentially as a measure of assay precision (Cummings *et al*, 2008) and under this definition 15 of 17 assays proved to be fit-for-purpose for use in clinical trials.

The success of this validation approach relies heavily on an accurate determination of the total error associated with each assay. Total error for an LBA is assumed to follow a normal distribution and consist of a systematic component (bias, measured as percent relative error (%RE)) and a random component (precision, measured as the coefficient of variation (%CV)) (Findlay *et al*, 2000; DeSilva *et al*, 2003; Findlay, 2009). If acceptance limits for the QCs are imposed at a mean value±a fixed CV (e.g. 15%) before total error is quantified (Shah *et al*, 2000) and the total error is subsequently established as being close to the fixed CV, then the acceptance limit is effectively set at 1 s.d. for the error in the assay. In this scenario, 2 out of 6 QCs and assays (33%) would be expected to fail randomly. To compensate, a 4 : 6:X rule is applied where only 4 out of the 6 QC are required to fall within the acceptance limit (X) and is an integral component of the fit-for-purpose approach (Lee *et al*, 2005, 2006). However, a problem arises when the total error is underestimated either by conducting too few experimental studies or by not taking sufficient account of batch-to-batch variability. Here an assay, which might well exhibit acceptable performance in the analysis of patients’ samples, may nevertheless fail at the pre-study validation stage due to adoption of inappropriate acceptance criteria (Findlay, 2009).

Evaluation of error and choice of acceptance limits was based on the results from 2–3 assays of eight replicate measurements of the QCs per run, which tended to weight the validation towards within-day/intra-assay precision. As this performance parameter often shows less variability than between-day/inter-assay precision, it is possible that the true level of imprecision was undervalued. Nonetheless, 15 of 17 assays did pass this stricter validation regime. However, an assessment of the errors associated with accuracy and bias, the systematic component in the total error model, was confounded by the fact that commercially available QC standards were used. These were provided by the manufacturer not at a nominal concentration together with a certificate of analysis but at high, medium and low concentration ranges, and thus could not be added at known concentrations. The issue of poorly characterised, or non-representative (recombinant proteins or peptide fragments), reference materials reconstituted in simple assay buffers to act as calibration standards and QCs in LBAs remains a perennial problem (Lee *et al*, 2006; Nowatzke and Wood, 2007; Findlay, 2009). As a consequence, most LBAs of biomarkers that use such reference materials can only be classified as producing relative quantitation in patient samples (Lee *et al*, 2005).

To counteract the possibility of underestimating error during a typical fit-for-purpose biomarker method validation, a revised strategy based on the data here is proposed for future studies. The most important point in the revised strategy is the adoption of a confidence interval (2 s.d.; 95% confidence interval) favoured in diagnostic biomarker QC (Westgard *et al*, 1994). In future, QC acceptance limits will be set provisionally at 2 s.d. on the basis of running four replicates on five separate assays (DeSilva *et al*, 2003). As before, three subsequent assays must fall within specification to consider the assay fit-for-purpose for use in clinical trials (Lee *et al*, 2006). Performance of the QCs should then be continually monitored, cumulative precision profiles plotted and acceptance limits modified until a ‘precision plateau’ – representing the total error – is reached. Acceptance criteria should then be fixed and changed only if batch-to-batch issues arise. It will be especially important to apply this revised approach as a biomarker progresses from research tool during early drug development, through proof of principle/concept during early phase trials, until becoming a fully qualified surrogate endpoint that can predict or report drug response in later phase trials, when QC issues become much more crucial (Lee *et al*, 2005; Cummings *et al*, 2008).

The stability of soluble protein biomarkers for analysis by ELISA assay is often assumed and studies of the effect of long-term storage of patient specimens before analysis (Aziz *et al*, 1999) are rarely conducted. In the good laboratory practice (GLP) environment, extensive characterisation of sample stability is required by the regulators (James and Hill, 2007), and these should be conducted in a matrix that mimics the characteristics of the test samples (Nowatzke and Wood, 2007). Analyte depletion or a matrix that is otherwise altered is not considered acceptable to the FDA. However, there are many reasons why protein instability occurs: bacterial contamination; protease/caspase degradation; denaturation; chemical instability (methionine oxidation, de-amidation, disulfide bond cleavage); folding/unfolding; insolubility; complex formation of a ligand with a soluble receptor and protein aggregation (Findlay, 2009; Mahler *et al*, 2009; Maity *et al*, 2009; Wu *et al*, 2009). Antibody-based assays, such as ELISAs, that depend on epitope recognition involving not only sequence but conformation are particularly susceptible to many of the above variables (Ling *et al*, 2007). Changes in protein conformation can manifest in either a decrease in concentration and apparent instability or an apparent increase in concentration (Nayeri *et al*, 2002; Cummings *et al*, 2007). Due to abnormalities in blood chemistry, including elevations in proteases and caspases, the stability profiles of protein biomarkers measured in cancer patients’ plasma are likely to vary significantly from those obtained in ‘cleaner’ matrices, especially buffers and even plasma from healthy controls (Deligezer *et al*, 2006; Findlay, 2009).

In this study, stability was assessed in three different contexts: recombinant protein in a surrogate matrix (porcine plasma/serum); endogenous analytes in healthy volunteer plasma and endogenous analytes in cancer patients’ plasma. Not surprisingly the greatest stability was observed in the surrogate matrix with recombinant proteins. In the healthy volunteers’ plasma, a marked decrease in PDGF-BB concentration occurred after 8 months at −80°C, whereas an equally substantial increase in KGF occurred over the same time frame. Smaller changes were also recorded in VEGF-A, PlGF and FGFb, but these were closer to our acceptance limit of a 25% change for instability. Platelet-derived growth factor is a dimeric protein held together by two disulfide bonds, which are essential for correct folding and stability of the protein (Ostman *et al*, 1993). The native monomeric sequence of the KGF has been shown to be unstable in plasma due to aggregation even at moderate storage temperatures (Hsu *et al*, 2006). Members of the FGF family have a short half-life *in vivo* due to denaturation at temperatures close to physiological (Zakrzewska *et al*, 2009).

In cancer patients’ plasma consistent increase in KGF concentration was also evident, even after 3 months of storage at −80°C, whereas a consistent decrease in PDGF-BB was recorded but only in platelet-depleted plasma. Sporadic changes (either increases or decreases in concentration) occurred with other analytes (VEGFR2, FGFb, HGF and VEGFR1) more randomly. These data would indicate that KGF is unstable in cancer patients’ plasma and that PDGF-BB in the absence of platelets (where the majority of PDGF is normally located) is also unstable in plasma. The sporadic instability observed with other analytes may be caused by biological variables – such as disease stage, age or treatment regimen – additional to the duration of storage at −80°C. It should be noted, however, that different analytical platforms were used to conduct the stability studies. Single-plex ELISA was used with the recombinant proteins and multiplex ELISA was used for both healthy volunteer and cancer patients’ samples. Therefore, some of the differences in stability profiles observed may be due to cross-platform variability.

In conducting stability studies, both statistical significance using the Wilcoxon signed-rank test and an increase or decrease greater than a predefined acceptance limit of 25% were required. This latter value was chosen as it is the default value for random error (imprecision) recommended in the fit-for-purpose approach to biomarker method validation (Lee *et al*, 2005, 2006). As a rule the imprecision in the assays used was below this value, both for the single plex and the multiplex. A notable exception was for PDGF-BB where this value could approach 30% (Backen *et al*, 2009). However, the fact here that there was statistical significance and a consistent change occurring in all five patients adds confidence to this result.

Platelets are known to sequester a number of angiogenesis-regulatory proteins including FGFb, PDGF-BB, VEGF, VEGFR1, Ang-1, HGF and SDF-1*α* (Nayeri *et al*, 2002; Brill *et al*, 2004; Klement *et al*, 2009). Thus, if the objective is to measure the ‘true’ level of free circulating protein it would be crucial to remove platelets and prevent the release of their contents before removal. Here a protocol is reported for effective removal of >90% of platelets that does not require recourse to a high-speed centrifugation step. The data also show that platelet removal should be performed before freezing plasma samples. Allowing blood to clot to harvest serum will also result in the release of angiogenesis analytes from platelets and haemolysis in plasma should be avoided. It is now evident that several circulating angiogenic cytokines are stored in platelets (Klement *et al*, 2009; Solanilla *et al*, 2009) and as platelet counts are elevated in cancer patients (Nash *et al*, 2002; Klement *et al*, 2009), there is perhaps a case for measurement of ‘free plus platelet-sequestered’ angiogenesis-associated factors (Klement *et al*, 2009). Whichever approach is taken, interpretation of the resultant data will require clarity on the inclusion or exclusion of platelets.

As most ELISAs are capable of only relative quantitation, one might expect different platforms, indeed even the same assay but sourced from different manufacturers, to yield discrepancies in the absolute concentrations measured in equivalent groups of patients (Cummings *et al*, 2008). Indeed, several previous cross-platform studies involving antibody-based ELISA technologies, including Endogen/Aushon Multiplex and singleplex ELISA R&D assays (as used in this present study), Meso-Scale Discovery (MSD) and Luminex beads, have shown that these differences can be as great as two- to five-fold (Urbanowska *et al*, 2006; Toedter *et al*, 2008; Chowdhury *et al*, 2009). Thus, cross-comparisons of antibody-based technologies show the true relative nature of the concentrations they report, and mandate the need to restrict analysis of clinical trial samples to a single platform. In this scenario the principal performance indicator becomes the sensitivity of the analytical platform to detect a meaningful (relative) change in biomarker concentration that is causally linked to a biological endpoint such as the effect of drug action. This ability will depend on the level of variation associated with the biomarker within the patient population as well as analytical issues. An assessment of within-day variation can be conducted by analysis of two separate samples collected from the same patient within a relatively short space of time, in the absence of drug treatment (Cummings *et al*, 2006). We have previously determined this value to be 13–14% for cell death biomarkers comprising different molecular forms of the protein cytokeratin-18 (Cummings *et al*, 2005, 2006). The ‘signal-to-noise’ values for the angiogenesis-associated analytes are the subject of ongoing investigation.

In summary, the studies reported here have highlighted the need to conduct assay validation and to address sample handling issues, such as stability and the impact of platelet removal, before commencement of clinical trials if such biomarkers are to yield information useful for drug development and patient care.

## Figures and Tables

**Table 1:** Fit-for-purpose method validation^a^ of 17 different ELISAs representing potential biomarkers of antivascular drugs using QC samples

	**Stage-1: Determine QC precision (% CV) (*n*=16 repeat analyses)** b				
	**QC concentrations**			**Stage-2: Set target acceptance limits for QCs Precision % CV**	**Stage-3: Do additional assays pass QC acceptance criteria Number of assays 2–3**
**Analyte**	**Low**	**Mid**	**High**		
CD105	4.83	4.53	8.58	20	Yes
VEGF-A	5.93	8.33	4.72	20	Yes
PlGF	5.68	6.07	5.91	20	Yes
VEGF-receptor-1 (R1)	10.1	12.3	10.9	20	Yes
VEGF-receptor-2 (R2)	5.29	3.15	4.21	20	Yes
Angiopoetin-1 (Ang-1)	2.67	2.98	2.25	20	Yes
Angiopoetin-2 (Ang-2)	7.73	3.17	10.4	20	Yes
TIE-2	4.77	5.04	6.47	20	Yes
VEGF-D	14.0	13.1	7.08	20	Yes
PDGF-BB	11.1	8.86	10.6	20	Yes
FGFb	3.30	3.53	3.75	20	Yes
IL-8	16.2	16.5	6.62	20	Yes
SDF-1*α*	4.40	4.96	6.25	20	Yes
HGF	15.8	9.39	7.10	20	Yes
Osteopontin (OPN)	6.91	7.52	6.83	20	Yes
KGF	17.6	11.0	5.00	20	No
VEGF-C	11.7	14.4	15.8	20	No

Abbreviations: CV=coefficient of variation; FGF=fibroblast growth factor; HGF=hepatocyte growth factor; IL=interleukin; KGF=keratinocyte growth factor; PDGF=platelet-derived growth factor; QC=quality control; VEGF=vascular endothelial growth factor.

a Fit-for-purpose assay validation was conducted as described in Figure 1 and essentially consisted of three stages. In the first stage precision in the QCs was determined experimentally. In stage-2, a target CV acceptance limit was set against which the performance of future assays was evaluated. Stage-3 required that 2–3 additional assays fell within these target CVs for the QCs, to consider the assay valid for analysis of clinical trials samples.

b CV was calculated as a percentage using the following formula: the standard deviation in the 16 replicates divided by the mean value of the 16 replicates, multiplied by 100.

**Table 2:** Duration of stability^a^ of recombinant standards of angiogenesis biomarkers spiked in porcine plasma (P) and serum (S) and stored at different temperatures

**Stability of angiogenesis biomarkers in porcine plasma (P) and serum (S)**								
	**Room temperature**		**4**°**C**		**−20**°**C**		**−80°C**	
	**P**	**S**	**P**	**S**	**P**	**S**	**P**	**S**
VEGF-A	1 d^a^	1 d	7 d	1 d	1 y	1 y	1 y	1 y
PlGF	<1 d	<1 d	7 d	<1 d	1 m	1 m	1 y	1 y
PDGF-BB	1 d	1 d	1 d	1 d	1 y	1 y	1 y	1 y
Ang-1							3 m	3 m
Ang-2							3 m	3 m
TIE-2	7 d	7 d	7 d	7 d	1 y	1 y	1 y	1 y
FGF-b	1 d	1 d	21 d	21 d	6 m	6 m	1 y	1 y
SDF-1*α*	7 d	1 d	7 d	7 d	1 m	1 m	1 m	1 m
IL-8	7 d	7 d	7 d	7 d	1 m	3 m	3 m	3 m
OPN	1 d	1 d	7 d	7 d	3 m	3 m	1 y	1 y
CD105							9 m	9 m

Abbreviations: Ang=angiopoetin; FGF=fibroblast growth factor; IL=interleukin; KGF=keratinocyte growth factor; OPN=osteopontin; PDGF=platelet-derived growth factor; VEGF=vascular endothelial growth factor.

a d=day/s; m=month/s; y=year/s. Recombinant proteins were spiked at a mid-range concentration and stored at room temperature, 4°C, −20°C or −80°C for different durations of time up to 12 months in the case of −80°C. At defined intervals samples were retrieved for analysis by singleplex with instability being defined as a significant reduction in concentration (⩾25%) occurring between two time points.
